# Gut Microbiota and Neuroplasticity

**DOI:** 10.3390/cells10082084

**Published:** 2021-08-13

**Authors:** Julia Murciano-Brea, Martin Garcia-Montes, Stefano Geuna, Celia Herrera-Rincon

**Affiliations:** 1 Department of Biodiversity, Ecology & Evolution, Biomathematics Unit, Complutense University of Madrid, 28040 Madrid, Spain; julia.murciano@estudiante.uam.es (J.M.-B.); garciamontesjosemartin@gmail.com (M.G.-M.); 2 Modeling, Data Analysis and Computational Tools for Biology Research Group, Complutense University of Madrid, 28040 Madrid, Spain; 3 Department of Clinical and Biological Sciences, School of Medicine, University of Torino, 10124 Torino, Italy; stefano.geuna@unito.it

**Keywords:** gut–brain axis, nutritional psychiatry, brain–bacteria communication, smart food

## Abstract

The accumulating evidence linking bacteria in the gut and neurons in the brain (the microbiota–gut–brain axis) has led to a paradigm shift in the neurosciences. Understanding the neurobiological mechanisms supporting the relevance of actions mediated by the gut microbiota for brain physiology and neuronal functioning is a key research area. In this review, we discuss the literature showing how the microbiota is emerging as a key regulator of the brain’s function and behavior, as increasing amounts of evidence on the importance of the bidirectional communication between the intestinal bacteria and the brain have accumulated. Based on recent discoveries, we suggest that the interaction between diet and the gut microbiota, which might ultimately affect the brain, represents an unprecedented stimulus for conducting new research that links food and mood. We also review the limited work in the clinical arena to date, and we propose novel approaches for deciphering the gut microbiota–brain axis and, eventually, for manipulating this relationship to boost mental wellness.

## 1. Introduction

Recent discoveries concerning the interaction between the microorganisms that inhabit our guts (or microbiota, mainly bacteria) and the central nervous system (CNS) have revolutionized neuroscience in the 21st century [1], making the microbiota–gut–brain axis (MGB) one of the most innovative research fields at the edge of multidisciplinary knowledge, with a clear translational impact. We now know that the gut microbiota may play a key role in the development and progression of certain neurological and neuropsychiatric conditions (such as Alzheimer’s disease (AD), autism spectrum disorder (ASD), depression, and anxiety) and that this influence is also bidirectional, as shown by the comorbidities of certain neural pathologies and intestinal dysbiosis [2] (such as metabolic syndrome, irritable bowel syndrome (IBS), Crohn’s disease, or alterations in the ratio of bacterial species of the microbiota itself detected in patients with depression). However, despite all these advances and new discoveries, the mechanisms underlying bidirectional microbiota–brain communication are still largely unknown. Crucially, the scientific community has focused on indirect pathways of connection through metabolites and signals that, in many cases, involve the immune and neurohumoral systems. Although this knowledge is necessary and studies provide relevant molecular data, there is a clear need for new methodologies and, most importantly, multi-integrative “top-down” perspectives that allow a holistic understanding of the bases and mechanisms of cellular interaction that underlie bidirectional microbiota–brain communication, in order to, eventually, transfer this basic scientific knowledge into biomedical solutions that are applicable to the great challenges of our society, such as the prevention or treatment of conditions related to mental well-being. 

In this review, this new exciting new scientific topic is tackled in an original way that covers, for the first time in the literature, topics ranging from the pathology and action mechanism (neuroplasticity) to suggested solutions (targeted nutrition) in the gut microbiota–brain bidirectional connection. We first set the stage by illustrating how the microbiota interacts with the human body and, specifically, the nervous system. Then, we discuss the translational context by addressing the two-way relationship between gut dysbiosis and major brain disorders. Finally, the perspectives of treating brain disorders through the microbiota, with a specific emphasis on food-mediated interventions (an approach that is likely to impact a large number of people worldwide), are outlined. The article is organized based on answering seven key scientific questions.

## 2. How Do the Microbiota and the Body Talk to Each Other?

The term microbiota refers to the enormous number of microbes (10–100 trillion) that colonize the human body [3]. The majority of microbial colonies are detectable in the gastrointestinal (GI) tract. However, microbes also colonize many other parts of the body, such as the skin and the genito-urinary tract [4]. The presence of this huge number of microbes, which greatly exceeds the number of human cells, makes it reasonable to assume that the microbiota can influence the structure and function of the body. This is even more reasonable if we further consider that the microbial genome (or microbiome) also largely exceeds the human genome of the host in size [5]. In fact, an increasing body of evidence is emerging showing that the microbiota indeed influences, both locally and systemically, the structure and function of all of the systems of the human body [6].

The microbial colonization of the guts of mammals occurs early in life, at the moment of birth, mainly through the vaginal canal. The human gut is composed of a balanced microbiota with two dominant (70–75% of the total) phyla, Bacteroidetes (e.g., *Bacteroides*) and Firmicutes (e.g., *Lactobacillus*, *Clostridium*, and *Enterococcus*) [7]. Other phyla, such as Proteobacteria, Actinobacteria, Fusobacteria, and Verrucomicrobia, are less represented. The composition of these microbes can then be influenced by different factors early in life (particularly the channel for delivery [8,9]) and as we age [10]. The host’s condition regulates the gut microbiota, including through diet, genetics, environment, exposure to drugs and antibiotics, and other lifestyle factors [11,12]. The large number of symbiotic microorganisms that compose the gut microbiota are closely connected with each other and with the host and play a key role in human health. Effects on the host’s metabolism are arguably the best-known direct effects of the gut microbiota. Non-digestible macronutrients (carbohydrates, proteins, and lipids) are digested by the gut microbiota to produce microbial metabolites and short-chain fatty acids (SCFAs) and yield energy [13]. In this way, gut microbes and their metabolites generate favorable effects on the host’s body, facilitating nutrient absorption, gut motility, and the integrity of the gastrointestinal epithelial barrier [14]. However, the microbiota supports various other biological functions and the physiology of the host [15] by modulating the host’s immune defenses [16], liver function [17], and metabolism [18], as well as affecting brain function [19,20].

## 3. Do the Gut Microbiota and Brain Talk to Each Other?

**Figure 1:**
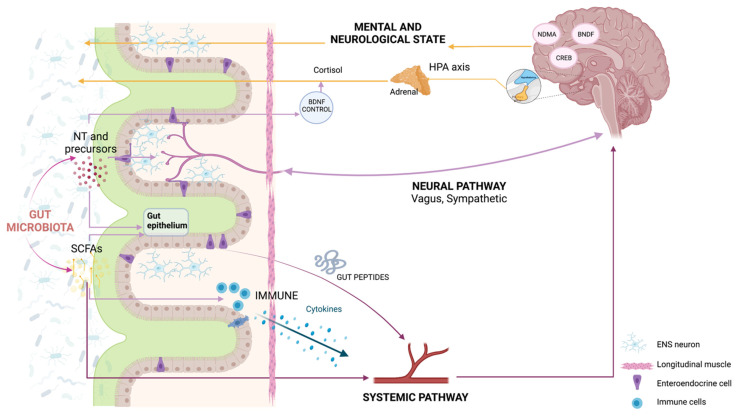
Communication pathways and mechanistic links between gut microbiota and brain plasticity. The microbiota–gut–brain axis comprises a complex bidirectional communication system mediated by neural (central nervous system, enteric nervous system (ENS), and branches of the sympathetic and parasympathetic systems), immune, endocrine, and humoral pathways. BDNF: brain-derived neurotrophic factor; CREB: cAMP-response element binding protein; HPA: hypothalamic–pituitary–adrenal; NMDA: N-methyl-D-aspartate; SCFAs: short-chain fatty acids. Created with BioRender.com (accessed on 25 July 2021).

Bidirectional communication between the gut and the brain has long been recognized (the gut–brain axis) [21,22]. Traditionally, the established pathways of communication encompass the neural pathway composed of intrinsic branches of the enteric nervous system (ENS); the extrinsic parasympathetic (mainly represented by the vagus nerve) and sympathetic branches of the autonomic nervous system (ANS); and the immune, endocrine, and humoral pathways [12] (Figure 1). The ENS is a large network with more than 100 million neurons of over 15 different cell types that innervate the gut [23]. The ENS is responsible for the regulation of gastro-intestinal processes and is connected to the CNS through the vagus nerve [23,24]. For this reason, studies on gut–brain communication initially mostly focused on the influence of the microbiota on the ENS and on how various diseases that affect the alimentary tract (e.g., IBS, inflammatory gut disorders, anorexia nervosa, and obesity) may dysregulate the gut–brain axis [24]. Evidence for the relevant influence of the microbiota on ENS development and homeostasis [25,26] and vice versa [27] has accumulated. It has been shown that the depletion of the microbiota negatively impacts the structure and function of the ENS [28,29], whereas the recolonization of adult germ-free (GF) mice, raised in the absence of microbiota, with a conventional microbiota has been shown to restore gastro-intestinal mobility [30]. Recently, it has been shown that that the enteric neuron-specific deletion of aryl hydrocarbon receptor (Ahr), a microbiota-dependent gene, negatively affects intestinal motility, pointing to the role of Ahr as a potential biosensor in ENS neurons [31]. From a phylogenetic perspective, it has been suggested that the ENS has evolved to orchestrate the responses from gut microbes and relay them to influence gut motility [32].

Whereas earlier studies focused on the relationship between the microbiota and the ENS, the existence of an intense cross-talk between the microbes in the gut and the whole nervous system has emerged over time, stimulating a new and very active area of research that is producing a growing body of experimental data from animal studies focused on the relation between the gut microbiota and the brain [33,34], suggesting that the gut microbiota is also a key mediator of gut–brain axis signaling in non-pathological conditions. One of the main pieces of evidence for the importance of the microbiota in brain function and development comes from GF studies in rodents. Using this strategy, focusing on deciphering what happens in the system when there are no microbes in it, many groups have demonstrated a variety of changes, including alterations of the myelinization in the prefrontal cortex, changes in volume and neurogenesis in the hippocampus, aberrant dendritic growth in limbic areas, blood–brain barrier permeability, and immature microglia [20,35,36]. This accumulating body of evidence for microbiota–brain communication, which involves nervous, endocrine, and immune signaling mechanisms, has led to the definition of the MGB model, and several putative mechanisms have been proposed to explain how the gut microbiota affects brain function and development.

Besides the widely known physiological actions in the host mediated by the microbiota, which mostly regulate immune and gastrointestinal function/metabolism, alterations in the microbiota can modulate neonatal brain development [37]; the host’s behavior [38]; and cognitive properties such as stress responses, anxiety, and fear extinction learning [35]. These dynamic and fine effects on brain function and behavior might suggest a closer relationship between these two biological entities. Recent studies have shown possible direct interactions of bacteria with brain function [39] and the ENS, leading researchers to re-think the microbiota–gut–brain axis in favor of a more specific bacteria–brain interkingdom communication [40,41,42]. Gut bacteria can influence the excitability and electrophysiological properties of enteric neurons through ion-channel-related actions [43,44]. Furthermore, it has been demonstrated that bacteria communicate with each other via some of the same mechanisms and algorithms used by neurons in the brain (ion-channel-mediated electrical and chemical signaling, which underlie computation) [40,45,46,47,48,49,50,51,52], performing cognitive tasks both as individual bacterial cells and as colonial super-organisms [53,54]. The rich behavioral and ion-channel- or bioelectricity-related repertoire of these two systems suggests that they, and the communication interface between them (which is increasingly being recognized as a crucial part of human physiology [41,55,56]), can be exploited to tackle fundamental questions of the bidirectional communication between neurons and bacteria.

## 4. How Are Gut and Brain Diseases Related to Each Other?

From a translational perspective, there is growing evidence that alterations in the gut microbiota may play a role in the pathogenesis and/or symptomatology of major brain disorders, emphasizing a clear need for more investigations to better understand the mechanistic links along the MGB axis. Initial clinical and preclinical studies on a likely relationship between the gut and brain were performed based on common observations of the high comorbidity of GI alterations in many neurodevelopmental, neuropsychiatric, and neurological diseases related to behavioral/motor abnormalities. A seminal demonstration of this inter-relationship was established in 1991 with Morgan’s work, which showed, for the first time, the improvement of symptoms in patients with hepatic encephalopathy (HE) after antibiotic treatment [57].

It is now widely known that the maintenance of a balanced gut microbiota (mostly in terms of composition) is critical for the correct functioning of gut physiology and the complex signaling of the MGB axis, thus impacting the host´s overall health. When the microbiota and/or its functions suffer an imbalance, which is referred to as “dysbiosis” [58,59], several systems can be negatively affected, ranging from the GI tract to the CNS. Likewise, alterations in the normal physiological state of the GI tract (such as increased intestinal permeability) or in the normal brain functioning can, in turn, induce the dysbiosis of the gut microbiota.

Autism spectrum disorder (ASD, including autism, Asperger’s syndrome, and pervasive development disorder not otherwise specified) is a complex neurodevelopmental disorder and, although its etiology remains unclear, comorbidities with GI symptoms (such as diarrhea and constipation) are conspicuously common and generally correlated with the severity of the neurobehavioral alterations [60], thereby making the gut microbiota a potential mediator of risk factors and an interventional target. At first, several studies demonstrated an altered composition of the gut microbiota in ASD patients, although these often provided contradictory or inconclusive results. With the advance of high-throughput sequencing techniques, a change in the structure of the gut bacterial community in ASD patients seems to be clear [61], with an elevated abundance of Proteobacteria (which is a major source of the antigen lipopolysaccharides (LPS) that promotes host inflammation) [62], a decreased proportion of *Bifidobacterium* [63] or *Prevotella* [64], and increased proportions of *Bacteroides* [65] and *Clostridium* [66,67]—both producers of propionate, which may aggravate the symptoms of ASD. It has been demonstrated that targeting the microbiota in ASD patients may significantly improve the symptomatology, such as through treatment with oral vancomycin for 8 weeks in ASD children [68] or with microbiota transfer therapy (MTT) [69], whose positive effects remained for two years after treatment [70]. 

Numerous recent reviews have focused on the correlation between an altered microbiota and neuropsychiatric disorders, with a special emphasis on depression and anxiety [22,71,72]. In schizophrenia, comorbidity with IBS [73], non-celiac gluten sensitivity [74], and the correlation between the amount of *Lactobacillus* and the severity of the symptomatology [75] also provide evidence of a possible connection to the MGB. Indeed, a reduction in the symptoms associated with the schizophrenia spectrum, such as delusions or disorganized behavior, has been associated with the use of a ketogenic diet [76], which could activate the auditory sensory gating deficit characterized in schizophrenia patients, as suggested in preclinical studies in DBA/2 mice [77].

Regarding neurological disorders, interesting recent studies have addressed the role of the microbiota in Parkinson’s disease (PD). Whereas Keshavarzian et al. [78] provided evidence that proinflammatory dysbiosis is present in PD patients and could trigger the inflammation-induced misfolding of α-Syn and the development of PD pathology, more recently, Bedarf et al. [79] showed significant differences in the colonic microbiota and the microbiota metabolism between PD patients and controls. These results were confirmed by Hill-Burns et al., who demonstrated that PD is accompanied by the dysbiosis of the gut microbiome in a large cohort of patients [80]. However, recent data show that gut dysbiosis plays a role in the occurrence of other neurological diseases, such as Huntington’s disease (HD) and motor neuron disease. Regarding HD, Kong et al. [81] showed the presence of a significant difference in microbiota composition in HD mice at 12 weeks of age. They specifically observed an increase in Bacteriodetes and a proportional decrease in Firmicutes. Interestingly, these differences were only detected in male HD mice. Regarding motor neuron disease, Fang et al. [82], in the case of amyotrophic lateral sclerosis (ALS), showed significant microbial changes between patients and controls, supporting the view that an imbalance in the intestinal microflora constitution has a strong association with the pathogenesis of ALS. However, Zhang et al. [83] showed, in an ALS mouse model, that butyrate administration restored intestinal microbial homeostasis, improved gut integrity, and prolonged life span. Furthermore, for Alzheimer’s disease (AD)—one of the brain disorders that places the highest health and economic burden on today’s society—evidence about the role of gut–brain crosstalk as a fundamental regulatory system in modulating neurodegeneration is emerging. In a Drosophila Alzheimer’s disease model, Wu et al. [84] showed that enterobacterial infection may exacerbate the progression of the disease by promoting immune hemocyte recruitment to the brain and neurodegeneration mediated by the TNF-JNK (tumor necrosis factor alpha-induced activation of c-jun N-terminal kinase) signaling pathway.

## 5. How Does the Brain React to the Environment?

Neuroplasticity can be defined as the ability of the nervous system to respond to intrinsic or extrinsic stimuli by reorganizing its structure, connections, and function. Plasticity refers to the capacity of a material to be physically malleable. Etymologically, plasticity derives from the Greek “plassein”, meaning to mold; thus, plasticity makes the brain malleable during its formation [85]. This plasticity is a key component for the neuronal and normal development of the central nervous system, involving modifications in responses to continuous environmental change. Regarding more cognitive aspects, neural plasticity can be defined as the ability to modify the functioning of neural circuits based on experience, thus affecting thoughts, feelings, and behavior [86]. Another remarkable aspect of plasticity is the fact that it occurs at various organizational levels, such as the nerve tissue, and can refer to neuronal, global, or synaptic plasticity. The understanding of the mechanisms of synaptic plasticity may offer important clues regarding the pathophysiological nature of neuropsychiatric disorders, pointing to new therapeutic approaches [86].

The variety of biological processes involved in neuronal plasticity ranges from neurogenesis to cell migration, along with changes in neuronal excitability or the modification of existing connections. In synaptic plasticity, one can differentiate between Hebbian plasticity, which involves a change in synaptic strength mediated by increasing or decreasing neuronal activity after the onset of stimulation (this being the case with long-term potentiation or LTP), and homeostatic plasticity, which constitutes a negative feedback loop in response to elevated neuronal activity. While Hebbian plasticity is involved in lifelong changes, homeostatic plasticity involves mechanisms such as the regulation of neuronal excitability or the stabilization of the total synaptic strength [85].

Although the study of brain plasticity has traditionally focused on the study of synapses, there are other approaches to this ability to modify the functioning of neural circuits that should be explored that could be related to the MGB axis, such as white matter plasticity or myelin plasticity (which has traditionally occupied a secondary role in the understanding of this behavior). This plasticity type offers another way in which the structure of white matter can be altered by experience [87]. Metaplasticity is another example of the modification of circuit functioning in which, although not expressed as an alteration of the regular synaptic transmission’s efficiency, there is a change induced by cellular activity itself. Processes such as *N*-methyl-D-aspartate (NMDA) receptor activation and postsynaptic upregulation are involved, as is synaptic plasticity. Studies on metaplasticity indicate that previous synaptic activation can leave a lasting imprint that affects the subsequent induction of synaptic plasticity [88].

In order to understand the experiments used to manipulate neuronal plasticity, we must first become acquainted with a series of markers of this phenomenon. There are different markers of plasticity, among which we highlight NMDA receptors, which participate in memory formation through the control of synaptic plasticity, or chemicals such as C-C motif chemokine ligand 11 (CCL11, also known as eotaxin-1) that are associated with both cognitive impairment and synaptic plasticity [89]. In addition, LTP and long-term depression (LTD), two of the main processes of synaptic plasticity, can be elicited by activating NMDA receptors, typically by the coincident activity of pre- and postsynaptic neurons. In studies with mammalian brains, considering both phylogeny and postnatal age, a common substrate for synaptic plasticity has been detected in the CA1 region of the hippocampus and in the superficial layers of the neocortex. This substrate involves the modification of excitatory synaptic efficacy according to the pattern or amount of NMDA receptor activated. Several studies have engaged in the search for common principles that can serve as the basis for a theory of synaptic modification [90]. Genetically, it is also known that the levels of certain transcription factors, such as the cAMP-response element binding protein (CREB), appear to influence activity-dependent plasticity [85]. Mechanistic links between the microbiota and neuroplasticity have mostly been revealed from GF model studies, which exhibit altered neurochemistry, neuroanatomy, and neurophysiology in the regions traditionally implicated in brain remodeling and cognition. The cellular and molecular pathways that seem to be affected in GF mice include alterations in the transcriptional profiles of excitatory neurons, glia, and other cell types in the prefrontal cortex, leading to alterations in neuronal function and fear extinction learning [35], lower brain-derived neurotrophic factor (BDNF) expression in the cortex and hippocampus [19,20], or a higher expression of immediate-early genes (Fos, Egr2, Fosb, and Arc) [91] and splicing factor genes [92] in the amygdala. Decreased levels of the neurotransmitter gamma-aminobutyric acid (GABA) in the hippocampus and neuroanatomical alterations such as reduced hippocampal volume and atrophies in pyramidal neurons [93] have also been found in GF mice. Interestingly, Darch et al. [94] have recently demonstrated sex-specific changes in the electrophysiological properties of the hippocampus in adult mice raised in the absence of a microbiota, with males being more affected, which is associated with dendritic signaling and LTP, suggesting an understudied role of the microbiome in individual differences in brain plasticity and cognition [95].

Brain plasticity can lead to an extreme degree of recovery through training and rehabilitation, which can modify and enhance these neuronal plasticity processes. Rehabilitation to achieve and maintain optimal physical, intellectual, psychological, and social functioning is one of the most successful therapies. For example, in the face of brain damage, it is known that there are certain critical time windows during which the brain shows a better response to the application of growth and plasticity-promoting agents [96]. In light of the brain’s ability to reorganize itself in response to intrinsic or extrinsic stimuli, microbiota-based approaches are emerging as a target for promoting neuroplasticity as both a diagnostic and therapeutic tool in a number of diseases of the nervous system, as reviewed above, such as ASD, AD, PD, depression disorders, addiction, and anxiety [97,98,99].

## 6. Can We Meet Our Own Microbiota?

To design effective therapies, the ability to model the states of microbiota with entero-types is vital [100,101]. Those entero-types need to represent different disorders or conditions and cannot be influenced by sex, nationality, age, or body mass index (BMI). In this context, a recent study showed at least three different entero-types in cases in which individuals in the same groups had similar reactions to a number of different drugs and diets [102]. The next exciting step would be to define biomarkers allowing a large share of inter-community variation to lose relevance and allowing us to focus on the elements of the microbiome shared between individuals with a given disorder [103].

Being able to associate biomarkers with disease stages is now a reality. The Firmicutes/Bacteroides ratio has been proven to indicate different stages of major depressive disorder, nonalcoholic steatohepatitis (NASH), ASD, PD, and AD. *Prevotellaceae* appears to be a biomarker for autism, and a low representation of *Prevotella* (linked to the long-term use of antibiotics) is associated with severe symptoms in PD [102,103,104,105].

The determination of the human microbiome is an ongoing effort from a large number of worldwide organizations (such as the National Institutes of Health-funded Human Microbiome Project (HMP; http://commonfund.nih.gov/hmp (accessed on 19 July 2021)), carried out over 10 years and two phases; the European-funded MetaHIT: Metagenomics of the Human Intestinal Tract (http://www.metahit.eu (accessed on 19 July 2021)); MICROB-PREDICT: microbiome-based biomarkers to predict the decompensation of liver cirrhosis and treatment response (http://microb-predict.eu (accessed on 19 July 2021)); ONCOBIOME: Gut OncoMicrobiome Signatures (GOMS) associated with cancer incidence, prognosis, and prediction of treatment response (http://www.oncobiome.eu (accessed on 19 July 2021)); and GEMMA: Genome, Environment, Microbiome, and Metabolome in Autism: an integrated multi-omic systems biology approach to identify biomarkers for the personalized treatment and primary prevention of autism (http://www.gemma-project.eu (accessed on 19 July 2021)) consortia), in an attempt to provide multi-omic data and other approaches to be adopted in future work on microbial dynamics, host responses, and microbial inter-relationships [106]. In spite of the difficulties in obtaining viable samples, new studies proving that most gut microbiota can indeed be culturable have been presented [107], and there is a promising future for using nanoscience and nanotechnology to directly measure and manipulate the microbiome ecosystem [108,109]. Culture-independent approaches such as 16S rRNA gene-based microbial profiling analysis are now standard, and adding a unique nucleotide barcode allows samples to be imported for fast analysis using a number of new pieces of software [110]. This technique is now being replaced by shotgun metagenomics, with the possibility of using metabolomic and metaproteomic analysis to identify which genes are expressed along with their regulatory networks. Novel next-generation sequencing (NGS) methods are leading the way to a strain-level reconstruction of genome sequences via metagenomic data, providing interesting taxonomic information about the strains [111]. As projects such as the HMP continue to expand, there is a pressing need for analysis tools. Big data will be very important in this field [112] and are used in the development of new software, such as QIIME, an open platform that analyzes datasets from users and compares them to those for other microbial communities [113]. New complex data are quickly surfacing, with new, exciting tools with which to understand them, representing a bright future for this field.

## 7. Can We Treat Our Own Microbiota to Promote Neuroplasticity?

**Figure 2:**
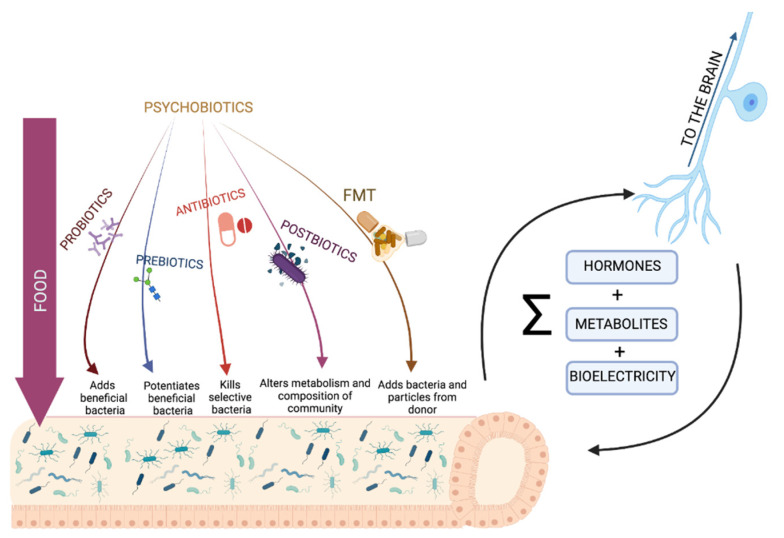
Microbiota–gut–brain–axis-targeted approaches. Food and psychobiotics [114] are the main exogenous factors whose impacts on the brain are mediated through gut bacteria. The pathways for bacteria–brain bidirectional communication, although still largely unknown, could involve hormones; metabolites; and, as recently proposed, bioelectrical signals [39,40]. FMT, fecal microbiota transplantation. Created with BioRender.com (accessed on 25 July 2021).

With the knowledge of the nature of the microbiota in different disorders, a great number of new therapies have been developed to target microbiota changes in a more specific and effective way (Figure 2 and Table 1). The term “psychobiotic” was originally coined by Dinan and colleagues in 2013 [114] to refer to probiotics (live organisms, mainly gut bacteria) and prebiotics (the fiber used in psychobiotics) with potential applications for treating psychiatric and mood disorders. This definition has recently been expanded, and any substance that exerts a microbiome-mediated psychological effect can now be considered a psychobiotic [41]. This includes more popular treatments, such as probiotics, prebiotics, and specific diets, as well as new and exciting techniques such as synbiotics, postbiotics, and even fecal microbiota transplantation (FMT). The intake of probiotics needs to be a daily-documented practice to be effective, as it is a complex method given the great differences between strains, but there have been very good results and extensive data on the health benefits and functions they bring [99,103,115]. Prebiotics are a new way to modify gut microbiota, inducing the growth of specific communities of bacteria that are pre-existent in our gut, with great results in recent studies. They are able to change the gut microbiota in a more general way rather than only improving one specific strain [116,117]. A way to ensure the colonization of the gut with a probiotic is to pair it with a prebiotic—known as a synbiotic.

Antibiotics were the first and most commonly used treatment for the gut microbiota; however, they are now starting to be seen in a different light in the context of the challenge that the increasing numbers of antibiotic-resistant genes in infants could bring in the future. However, there is a large body of evidence regarding the health improvements they confer for different disorders. FMT is emerging as a promising strategy that is especially relevant for behavioral aspects of ASD, with additional advantages derived from the accessibility of its procedure [103,105]. However, viable bacteria are a small fraction of the total transferred combination, with some risk being involved in the process [98]. Some of the most innovative practices use non-viable bacterial parts or metabolites, such as postbiotics, with interesting potential given their higher shelf-lives and reduced risks. One particular kind of postbiotics, named parabiotics—“heat-killed probiotics”—has recently shown great results. Heat-killed *Lactobacillus paracasei* PS23 has been shown to reverse hippocampal and prefrontal cortex abnormalities in dopamine levels in a depressive phenotype [103].

In order to develop new treatments, we need a deeper understanding of the signaling and a closer look at the way those drugs and treatment work. On this topic, proton pump inhibitors (PPIs) are being studied to investigate their proven effects on the gut microbiota, leading the way to new exciting psychobiotic approaches without the need to add antibiotics [124]. Furthermore, PPIs are now being associated with the risk of dementia and AD [125]. Functional MRI (fMRI) has proven to be very useful for helping practitioners to gain a better understanding of the underlying signaling by proving associations between external treatment and behavioral outcomes, such as the inter-relationship between the infusion of fatty acids and a reduction in the neural and behavioral responses of sadness in humans [126], or the intake of fermented milk products with probiotics by women and a reduction in reactivity in brain regions associated with emotional attention tasks [127].

The integrated multi-omic analysis (including 16S analysis, transcriptomics, proteomics, and metabolomics) of both the microbiota and host cells holds the potential to bring new insights and decipher the functional mechanisms of microbiota–host interactions. Analyzing the RNA transcripts in human mucosa has shown that the intake of known probiotic strains of *Lactobacillus* (*L. acidophilus, L. casei,* and *L. rhamnosus*) induces a differential expression of gene-regulatory networks and pathways [128]. The proteomic analysis of *Bifidobacterium longum* subsp. *infantis* in response to different prebiotics has revealed a clear association between the sugar ATP-dependent transport system and the consumption of galacto-oligosaccharides (GOS), fructo-oligosaccharides (FOS), and human milk oligosaccharides [129]. However, metabolomic analysis might provide interesting novel knowledge about the underlying mechanisms of the influence of the microbiome on the development of neurodegenerative and neuropsychiatric disorders in humans, as demonstrated by abundant evidence pointing to specific gut metabolites linked to certain CNS disorders [130,131,132].

In parallel with the accumulating new analytical data, the approaches to treating the microbiota include a broader spectrum of molecular and cellular targets [133]—for example, manipulating the activity of Toll-like receptors (TLR), as the gut microbiota produces several TLR ligands that have been linked to the development of diseases associated with an inflammatory status [134], or using probiotics in an attempt to regulate tryptophan and serotonin metabolism [135] are being intensively researched. Owing to the microbiota´s plasticity, lifestyle interventions (mainly in diet, exercise, and stress) are being increasingly recognized for their impact on physical and mental health, through gut–microbiota-mediated actions [136]. Exercise has been demonstrated to especially have an impact on Firmicutes and Actinobacteria [137,138], which include the *Lactobacillus* and *Bifidobacterium* genera, respectively, and to induce changes in the diversity and microbial production of SCFAs, specifically butyrate [139], which improves anxiety scores [140].

## 8. Can Targeted Nutrition Harness the Gut Microbiota and Promote Neuroplasticity?

The most consistent treatment for our gut microbiota is the one in which we engage the most often: food. Nutritional status and diet composition are two key factors that are reasonably easy to manipulate that determine the status of the gut microbiota [99,103,119,141]. Society’s interest in healthy eating has flourished in recent years, with increasing amounts of the literature covering the topic [142] and with an increasingly broad interest in nutritional psychiatry [143] and psychobiotics [144].

**Figure 3:**
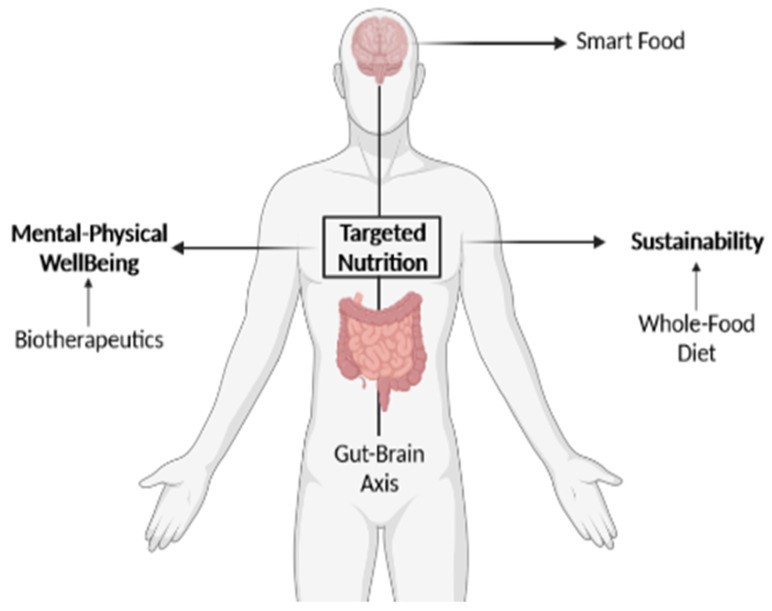
Targeted nutrition as a science-driven solution that directly addresses the SDGs “Good Health and Well-Being” and “Responsible Consumption and Production”. The conceptual representation of the beneficial effects of promoting a healthy, personalized, and sustainable diet targeting the microbiota–gut–brain axis to promote mental wellness (or *smart food*). Created with BioRender.com (accessed on 20 July 2021).

Long-term dietary patterns, such as a high consumption of protein from an unbalanced source, whether animal or vegetal, and low fiber intake, can affect the composition of the gut microbiota [130,141]. Prebiotics are naturally found in vegetables, grains, and fruits. Whole-food diets have experienced a dramatic reduction in prevalence in the Western-style diet (characterized by high fat and sugar intake), leading to the prevalence of metabolic syndromes [103,141] and other non-communicable, yet interrelated neurological/neuropsychiatric diseases. The Western diet leads to a change in the Bacteroides/Firmicutes ratio and to an increase in the relative abundance of *Collinsella,* both of which are common features in obese people [141]. Conversely, the Mediterranean diet, based on the daily intake of fruit and vegetables, whole grains, legumes, nuts, fish, white meats, and olive oil, has been shown to have important benefits for mental health, and is associated with a lower risk of developing AD, depression, and cognitive impairment [103,141]. The mechanism of action is not yet well-defined, particularly for prebiotics, and how alterations in gut bacteria can affect these brain functions and behaviors has not been fully explained in the vast majority of studies related to treatments [119]. This extends to other methods such as FMT, with an insufficiently defined mechanism of action, which is presumably very complex. No two donors or receptors are equal, so to truly make FMT a viable way to treat illness, there is a need to understand the mechanism of FMT’s effects step by step to understand what allows it to work on some people and not work on others [121]. Added to this are the very present strain-dependent properties of psychobiotic treatments; without a deeper understanding of the communication between the brain and bacteria, we depend on trial and error [103]. Even when using the same exact species, other factors such as age have appeared to be important, to the point of the treatment against a *C. difficile* infection not being efficient in elder patients [145]. Concerning safety, without knowing the exact paths those treatments follow, compromised patients, such as patients who are immuno-suppressed or suffering from malnutrition or cancer, cannot be considered for this kind of treatment [120]. A deep science-driven understanding of the actual value of the diet for physical and mental well-being, promoting smart food choices, would open a fascinating avenue for the development of personalized and microbiota-targeted nutritional approaches. There is, therefore, a need for studies that resolve the lack of knowledge and exploitation of our own natural resources: the interaction between the gut and brain and how we can harness this through sustainable nutrition (Figure 3), simultaneously referring to the Sustainable Development Goals (SDGs; UN, 2015, http://sdgs.un.org/goals (accessed on 20 July 2021)) numbers 3 (Good Health and Well-Being) and 12 (Responsible Consumption and Production).

Translation from animal to human models is also vital. GF is the most used model in those trials, which narrows down the diversity in the human gut microbiota in a critical way. Furthermore, diets and microbiota compositions are very different in different species, leading to a need for human models [146]. The idea of “deep phenotyping” has emerged for the longitudinal study of the genome, proteome, metabolome, and microbiome and the modulation of those factors in the same individual [147,148]. Focusing on obtaining causal relationships between certain changes in those variants could identify changes from wellness to disease and help in preventing or even reversing this [147]. The Human Phenotype Ontology (HPO, http://hpo.jax.org (accessed on 24 July 2021)) project uses the analysis of phenotypic abnormalities to push precision medicine with computational deep phenotyping, with the intention of identifying disease etiologies [148]. On that note, the prospect of finding microbe–phenotype relationships would be a huge step in the right direction when aiming to identify causal microbes [149].

In this context, in vitro gut fermentation modeling, such as the Polyfermentor Intestinal Model (PolyFermS) [150], represents a useful tool for functional testing and screening analysis. The objective of these in vitro platforms is to recreate the known environment of an individual in an experimental setting, and thus in controlled conditions, thus providing the ability for direct translation to medical hypotheses and overcoming ethical concerns and difficulties regarding body sampling [151]. The search for biomarkers and causal microbes is mostly happening in Europe, the United States, and China, thus information from Africa, South America, and southeast Asia is lacking. There is a need to define what a healthy microbiota looks like, which entails looking at all the differences not caused by illness from everywhere around the world, including in different environments and diets [152].

## 9. Concluding Remarks

The literature overview provided in this paper clearly supports the view that the bidirectional communication between the gut microbiota and the brain raises the potential for targeting the microbiome in the development of novel approaches to promote mental well-being.

Can we further decipher the bidirectional communication between the gut microbiota and the brain to better understand how to manipulate it to promote neuroplasticity? Communication between neurons and bacteria is an increasingly studied topic in biomedicine and neuroscience, especially owing to the effects of the gut microbiota on brain function and behavior. Interactions between neurons and bacteria can be mediated by a variety of signals (biochemical signals; neurotransmitters; hormones; and, as is less broadly known, bioelectrical signals). In 2015, Suel´s group [48,153] demonstrated that bacteria communicate with each other within a biofilm using ionic currents, in a similar way to neurons in the brain. Furthermore, these slow ion floes are also used to communicate between biofilms, demonstrating that electricity could be a medium for communication that has been highly conserved during evolution [40]. Boosting the unique properties of the bioelectrical signals as mediators of the neuron–microbiota bidirectional communication might open new perspectives in this highly interdisciplinary field [39,154]. It is thus time to re-consider and combine efforts from different disciplines in an attempt to decipher the mechanisms underlying the brain–bacteria interaction in order to design novel interventions to prevent and treat conditions that might be triggered by this dysregulation. We foresee the implementation and launching of innovative strategies (such as digital tools or devices) designed to translate gut–brain science into a reality in our new context, with emerging technologies oriented to brain–machine interfaces and sensor biohacking as key players.

In conclusion, there is an increasing body of evidence that microbiota-targeted interventions can be designed with the goal of sustaining neuroplasticity. In fact, not only have many pieces of evidence been provided showing that the manipulation of the microbiota can promote neuroplasticity, but several possible mechanisms of action have also been put forward to provide a mechanistic explanation of how the microbiota’s therapeutic effect is mediated.

## Figures and Tables

**Table 1:** Summary of the impact of different types of psychobiotics [41] on cognitive, emotional, and neural variables.

Therapy	Results	References
Probiotics	Intake of Lactobacillus reuteri increases oxytocin levels, improving social behavior. Interventions with *Bifidobacterium longum, L. helveticus,* and *L. acidophilus* result in a general improvement in depression, anxiety, and stress. Furthermore, multi-strain probiotics are relevant, such as a multibiotic containing *Streptococcus thermophilus* (2 strains), *L. bulgaricus, L. lactis, L. acidophilus, L. plantarum, Bifidobacterium lactis,* and *L. reuteri*, which have an anxiolytic effect, or the combination of three strains—*L. acidophilus, L. casei*, and *B. bifidum—*which decreases scores of depression. Intake of a multibiotic containing *L. acidophilus, L. rhamnosus,* and *B. longum* improves conditions in autism spectrum disorder (ASD) cases. The cocktail VSL#3, including eight Gram-positive bacterial strains, has been proven to decrease microglial activation and to change the expression of genes linked to inflammation and plasticity in the brain. *B. longum 1714* has also been found to have a positive impact on cognition in mice. In cases of *Trichuris muris* infection, intake of *B. longum* decreases anxiety-like behavior induced by the parasite, and treatment with *L. rhamnosus* decreases those behaviors in mice. Intake of probiotics containing *Lactobacillus* prevents the memory deficits induced by stress in *Citrobacter rodentium*-infected mice. Treatment with Bifidobacteria reverses behavioral problems in rats with maternal separation depression, restoring levels of noradrenaline and normalizing the immune response.	[99,103,118,119]
Prebiotics	Interventions with a galacto-oligosaccharide mixture (B-GOS) have led to improvements in behavioral problems in children with ASD, to decreased anxiety levels in an irritable bowel syndrome cohort, and to an overall reduction of cortisol awakening responses in healthy controls. Intake of isolichenan (α-glucan from the lichen *Cetrariella islandica*) reverses ethanol-induced memory impairment in mice. Supplementation of diet with a mixed polysaccharide product improves cognitive function in adults. Intake of arabinoxylan from the yeast *Triticum aestivum* and β-glucan from barley preserves memory in mice with vascular dementia.	[103,119,120]
FMT	Fecal microbiota transplantation (FMT) from healthy individuals improves several behavioral aspects of ASD. Hepatic encephalopathy (HE) patients who received an FMT from a healthy donor experienced a decrease in HE episodes along with an increase in their cognition.	[103,121]
Antibiotics	Germ-free mice treated with antibiotics during the adolescent period show reduced anxiety and improved cognition. When those mice reach adulthood, the tryptophan metabolism is altered, with significantly reduced brain-derived neurotrophic factor and oxytocin expression. Treatment with the broad-spectrum antibiotic vancomycin leads to improvements in behavioral problems associated with ASD. Treatment with ampicillin blocks memory deficits generated by phencyclidine (PCP), a common drug used to treat schizophrenia-like syndromes. Intake of antibiotics that target *Helicobacter pylori* improves clinical outcomes of Parkinson’s disease. Antibiotic therapy that alters the gut microbiota can be used to potentiate the action of antipsychotics in patients with schizophrenia.	[99,103,119,122]
Postbiotics	Intake of a short-chain fatty acid (SCFA) combination—a mixture of acetate, propionate, and butyrate—has anxiolytic effects on stressed mice. Gut peptides have a well-established role in influencing behavior, stress, anxiety, and depression. Heat-killed *Lactobacillus paracasei* PS23 reverses the reduction in dopamine levels in a corticosterone-induced depressive phenotype. Reports on the use of SCFAs in animal models show symptom relief for multiple sclerosis, decreasing inflammation and demyelination in the brain.	[103,123]

## Abbreviations

AD: Alzheimer’s disease; Ahr: aryl hydrocarbon receptor; ALS: amyotrophic lateral sclerosis; ANS: autonomic nervous system; ASD: autism spectrum disorder; BDNF: brain-derived neurotrophic factor; CNS: central nervous system; CREB: cAMP-response element binding protein; ENS: enteric nervous system; FMT: fecal microbiota transplantation; FOS: fructo-oligosaccharides; GABA: gamma-aminobutyric acid; GF: germ-free; GI: gastrointestinal; GOS: galacto-oligosaccharides; HD: Huntington’s disease; HE: hepatic encephalopathy; HPA: hypothalamic–pituitary–adrenal; IBS: irritable bowel syndrome; LTP: long-term potentiation; MGB: microbiota–gut–brain axis; NASH: nonalcoholic steatohepatitis; NMDA: N-methyl-D-aspartate; PD: Parkinson’s disease; PPIs: proton pump inhibitors; SCFAs: short-chain fatty acids; TLR: Toll-like receptor.
